# Simple Electroosmotic Pump and Active Microfluidics with Asymmetrically Coated Microelectrodes

**DOI:** 10.1002/smsc.202300026

**Published:** 2023-07-09

**Authors:** Jun Liu, Jiawei Chen, Jia Dai, Jinyao Tang

**Affiliations:** ^1^ Department of Chemistry The University of Hong Kong Pokfulam 999077 Hong Kong

**Keywords:** electroosmosis, microfluidic pumps, micromixers

## Abstract

Electroosmotic pumps can deliver liquid without moving parts, making them suitable for microfluidic and lab‐on‐chip systems. Previously, alternating current electroosmotic pumps were constructed using pairs of coplanar asymmetrical interdigitated microelectrodes on the same substrate. In this work, a simpler micropumping system is developed, separating the electrodes on two substrates and breaking the symmetry by half‐depositing electrodes with 3D microstructures. Numerical simulation models of the pumping system and experimental velocity profiles are used to explain the fluid motion mechanism and structure‐dependent pumping performance. In addition to its efficiency and simplicity, this new pumping system also allows for the creation of a microvortex device and an active microfluidics device. This scalable micropumping system provides a way to pump liquids at microscopic or macroscopical scale in complex microfluidics systems.

## Graphical abstract

A surprisingly simple electroosmotic micropump is developed by glancing‐angle deposition of gold on 3D microstructures and pairing with a planar counter electrode. The micropump operates with low‐frequency low‐voltage AC power and can be easily integrated to generate complex flow fields, including microvortex and active microfluidics circuits.

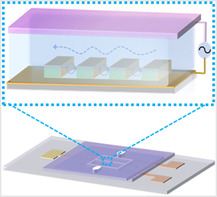

## 1 Introduction

Flow control at the microscale is critical in many important applications, including lab‐on‐chip devices, microanalysis systems, and micro liquid forced cooling systems.^[^ ^1^, ^2^, ^3^, ^4^, ^5^, ^6^ ^]^ However, traditional mechanical pumps that rely on external actuators and valves to regulate flow^[^ ^7^, ^8^ ^]^ can be difficult to miniaturize for integrated portable flow control devices. Electrophoretic micropumps offer a promising alternative to mechanical pumps in microfluidics. These devices utilize the principles of electroosmosis to drive fluid flow in microscale systems, providing precise and controlled fluid handling in small volumes without the need for external actuators.^[^ ^9^, ^10^ ^]^ Electrophoretic micropumps can be miniaturized for integration into portable flow control devices, making them an important tool for advancing microfluidics in a broad range of applications.^[^ ^11^, ^12^, ^13^, ^14^ ^]^

On the other hand, as the size of microfluidic device shrinks, the pressure demand to maintain the appreciable flow rate soars. This suggests that pressure‐driven fluidics becomes impractical as the device size decreases to nanometer scale. To overcome this limit, many other methods have been developed to manipulate and control the microscale flow, primarily utilizing discontinuous surface stress on an active surface.^[^ ^15^ ^]^ These methods can be generated by thermal gradient,^[^ ^16^, ^17^ ^]^ magnetohydrodynamics,^[^ ^18^, ^19^ ^]^ electrohydrodynamic,^[^ ^20^, ^21^ ^]^ or by surface modification.^[^ ^22^ ^]^

In microfluidics, two types of electrophoretic micropumps are commonly used: direct current (DC) electroosmotic pumps and alternating current (AC) electroosmotic pumps. DC electroosmotic pumps are the most basic type of electrophoretic micropump and utilize a constant DC voltage to drive fluid flow.^[^ ^15^, ^23^ ^]^ While these pumps are simple to use and widely available, they have some limitations, such as the high voltage requirement and the potential for electrochemistry reactions that can affect the fluid's quality.

To overcome this limitation, another electrophoretic micropump type, the alternating current (AC) electroosmotic pump, is developed. Unlike direct current (DC) electrophoretic micropumps, AC electroosmotic (ACEO) pumps use an alternating electric field to drive fluid flow.^[^ ^24^, ^25^, ^26^, ^27^, ^28^ ^]^ This allows for the use of lower voltage, reducing the potential for electrochemical reactions and increasing the stability of the fluid flow. To break the symmetry, the ACEO pump usually utilizes asymmetrically arranged interdigitated microelectrodes, where pairs of microelectrodes are deposited on the same substrate. The different width or height of the microelectrode results in an asymmetrical distribution of electric field,^[^ ^25^, ^26^ ^]^ generating unidirectional flow. However, this coplanar geometry requires high‐precision lithographic patterning and is potentially susceptible to failure due to the proximity of two electrodes, which limits the scalability of the device.

In this work, we designed a much simpler electric pumping device based on a noncoplanar ACEO. Two electrodes are deposited separately on oppositely faced substrates, while an asymmetric 3D microstructure is used to break the symmetry. This device greatly simplifies the fabrication, making it suitable for scale‐up applications and offering more design flexibility to generate complex flow fields. In particular, the micropump is prepared by depositing asymmetric metal lines with a glancing angle and paired with a counter ITO glass electrode. The directional flow can be created by applying low AC voltage across the oppositely faced electrodes. The flow motion in the channel is the result of the bending electric field, as confirmed by numerical simulation and experiment. To show the flexibility of this design, we demonstrated a microvortex generator and a three‐channel microfluidics pump by integrating multiple pumping segments in a microfluidic device.

## 2 Fabrication and Pumping Mechanism of Noncoplanar Micropumping

**Figure 1:**
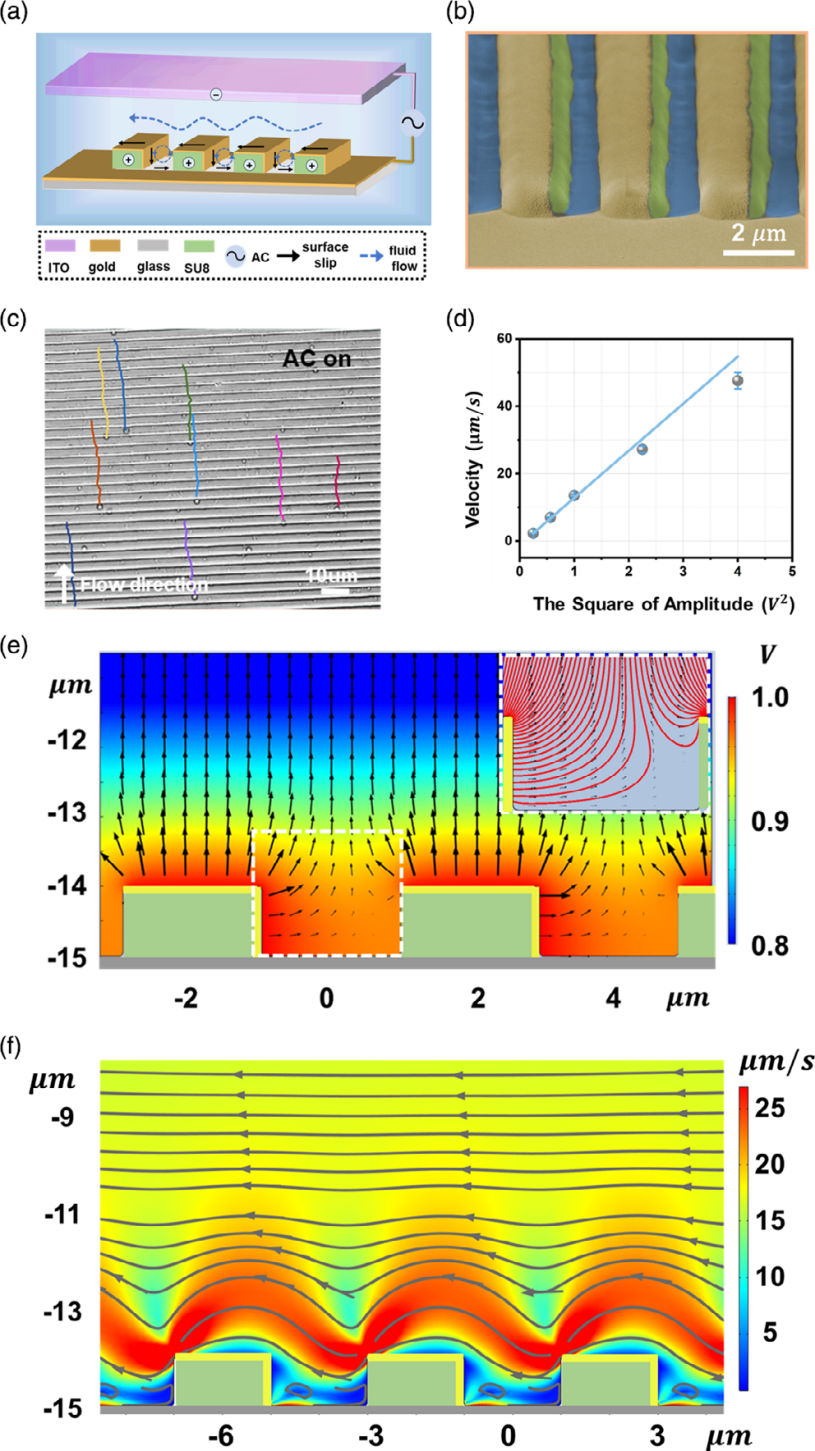
Schematics of ACEO micropump device and mechanism. a) Illustration of the micropumping device containing line microstructures with asymmetric metal coating. b) False‐colored SEM image of SU8 lines deposited with gold. The yellow, green, and blue represent the gold, SU8, and glass, respectively. c) Tracer particle trajectories over 2 s duration with applied AC voltage, calculated from Video S1, Supporting Information. d) The linear relationship between pumping velocity and square of applied peak voltage. The error bars of velocity represent the 95% confidence interval of mean velocity with at least 100 speed points. e,f) Simulated electric field (e) and flow field (f) near the electrode surface on the 3D electrodes. Inset of (e) is enlarged from the dashed square area, and the red line is the electric field line, indicating the bending electric field around the electrodes.

As illustrated in **Figure** **1**a, the pumping device is composed of an asymmetric nanostructured metal line electrode and a counter‐planar ITO electrode. The detailed fabrication procedure is described in the Supporting Information Device Fabrication and Characterization section. In brief, the periodic lines with 1 μm‐thick SU8 photoresist are first patterned lithographically on the glass substrate with a tunable width and spacing distance. Then, these SU8 lines are coated asymmetrically with gold by glancing‐angle deposition (GLAD),^[^ ^29^ ^]^ which only coats one side of the SU8 sidewalls, leaving the glass substrate uncoated (see Figure 1b). An ITO glass is thermally bonded with the patterned substrate with a parafilm spacer (30 μm thick), which forms the microfluidic channel.

The micropump is first demonstrated on the device with 2 μm line width and 2 μm gap distance. Silica microparticles are used as tracers to indicate the flow. Upon applying 1 V_peak_ sinewave potential, a unidirectional flow originating from the surface electroosmotic slip is observed, as shown in Figure 1c, while the planar device with the same width and gap does not show directed flow as expected. The generated flow motion is an electrokinetic osmosis flow,^[^ ^30^, ^31^ ^]^ which is proportional to the square of the applied field ${\left|E\right|}^{2}$ (see Figure 1d).

Due to the electrode geometry, we attribute the unidirectional electroosmotic flow to the electric field bending near the electrode.^[^ ^32^ ^]^ The simulation result by COMSOL Multiphysics reveals the bending electric field line (see Figure 1e). During the positive cycle of the applied AC potential (see Figure S2d, Supporting Information), the polarities of the bottom asymmetrical gold electrodes are positive, with the electric field starting from the bottom electrodes. This creates an electric double layer (EDL) parallel to the electrode that is dominant in anions. The tangential component of this electric field along the surface induces the electroosmosis flow by interacting with the EDL of the fluid induced by the applied AC potential, which subsequently leads to bulk fluid motion via the viscous drag.^[^ ^24^ ^]^ Under an alternating field, the polarity of EDL switched synchronously with the applied field, which resulted in unidirectional electroosmotic flow in both positive and negative half of the AC field. This flow field can be visualized by the simulations of time‐average velocity around the electrode, as shown in Figure 1f, which confirms the proposed pumping mechanism.

## 3 Structure‐Dependent Pumping Performance

**Figure 2:**
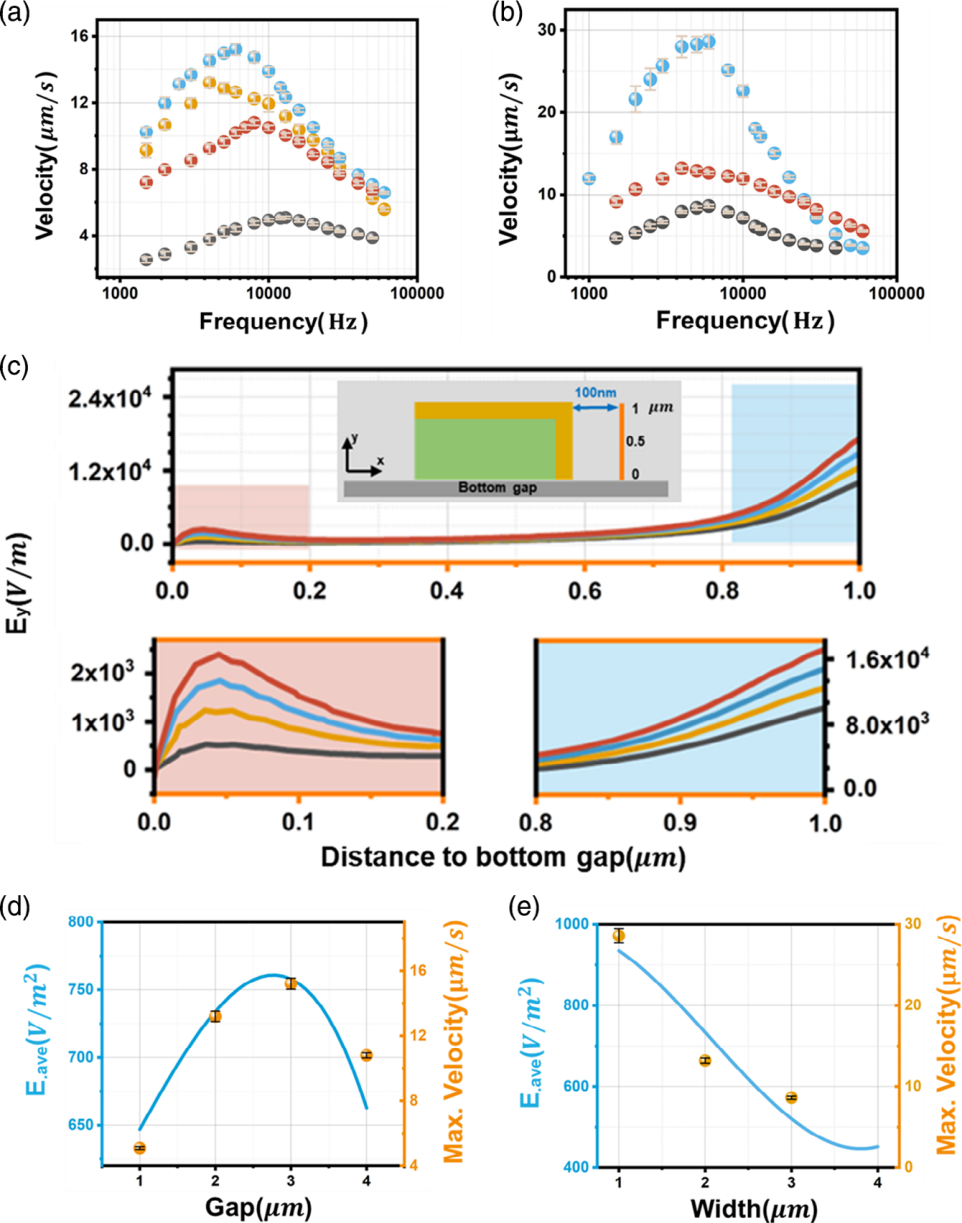
Pumping performance under different electrode structures. a) Experimental pumping velocity for different gap values. The blue, yellow, red, and black color represents 3, 2, 4, and 1 μm gap, respectively. *w* ≈ 2 μm. b) Experimental pumping velocity for different width values. The blue, red, and black colors represent 1, 2, and 3 μm width, respectively. The *g* ≈ 2 μm. c) Electric field strength in the direction paralleled with the side wall surface under different gap distance, denoting the $E_{y}$ (*y* component of the electric field) at the position of the orange line (insert schematic of (c)). The red, blue, yellow, and black colors represent 4, 3, 2, and 1 μm gap, respectively. d,e) The *E*_ave_ (blue line) and maximum experimental velocity (yellow sphere point) for devices with different gaps (d) and different widths (e). The maximum velocity is from (a,b).

In our electrophoretic micropump that uses a 3D microelectrode, the pumping flow is primarily generated by a local, distorted electric field around the electrode. As a result, all parameters that impact the electric field surrounding the electrode will be critical in determining the pumping efficiency. A series of devices (see Figure S2b, Supporting Information ) with varying gap distances (from 1 to 4 μm) and electrode widths (from 1 to 3 μm) were fabricated to study the structure–function relationship of the ACEO pump. As shown in **Figure** **2**a,b, the flow velocity is measured with 1 V_peak_ amplitude applied voltage and frequency range from 1 K to 100 kHz for deionized water. The maximum pumping speed is obtained at ≈10 kHz and goes to zero at high‐ and low‐frequency limits for all devices. If a simple linear approximation is considered, the velocity of ACEO is proportional to the product of the tangential electric field and the charge density in the EDL.^[^ ^24^ ^]^ The electric field in the bulk solution is frequency dependent because of the electrode polarization.^[^ ^33^, ^34^ ^]^ At low frequencies, the potential across the bulk solution is tiny as most of the applied voltage relaxes across the EDL, which leads to small tangential electric field at the EDL, resulting in low velocity. On the other hand, at high frequency, the surface charge density is low as the potential across the EDL drops,^[^ ^35^, ^36^, ^37^ ^]^ leading to low pumping speed.

With the fixed gold line width (2 μm), the spacing distance is varied from 1 to 4 μm. As shown in Figure 2a, the optimal spacing is 3 μm, which can be rationalized by considering the slip flow unit density. For a single pumping unit (one pair of electrodes and spacing), the tangential electric field around the side wall surface of the electrode is stronger with gap distance, which was confirmed by the simulation results. As shown in Figure 2c, the electric field ($E_{y}$) paralleled with the side wall surface of microelectrode at the position of 100 nm (the approximate thickness of the double layer in deionized water at low voltage)^[^ ^25^, ^38^ ^]^ away from the surface is increasing with spacing distance from 1 to 4 μm, which leads to enhanced pumping speed. On the one hand, sufficient spacing is needed to create unidirectional pumping while the spacing should not be too large to ensure higher pumping unit density. On the other hand, as the electric field bending is concentrated on the metal line edge, smaller linewidth is preferred for higher pumping efficiency (Figure 2b). The device with 1 μm width and 2 μm gap achieved the best pump performance during these tests. When compared by the pumping speed over square of electric field, its velocity is comparable to or better than that of state‐of‐the‐art ACEO pumps in most previous works, as shown in Table S2, Supporting Information.

**Figure 3:**
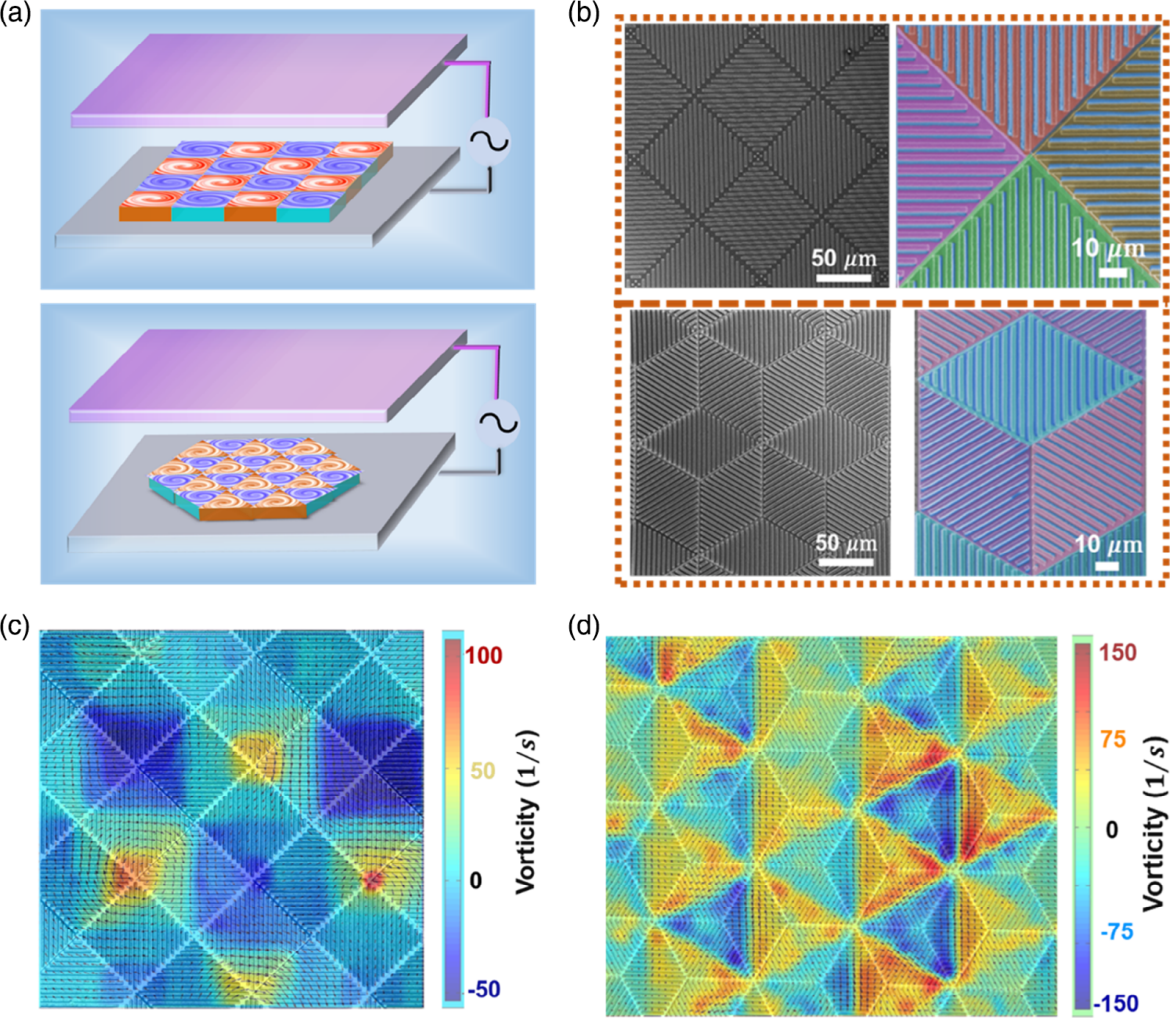
Demonstration of macroscopic vortex generator device. a) Illustration of microvortex device with square (upper) and triangle (bottom) layout. b) SEM images of the square (top) and triangle (bottom) layout microvortex devices. The false‐colored SEM images (right) show the different deposition directions of the gold electrode, where the blue color represents the bottom substrate and other colors represent the different metal deposition directions. c) Microscopy images of microvortexes array covered by the experimental vorticity field, calculated from Video S2, Supporting Information.

Hence, we propose a variable combining the electric field strength and the density of pumping unit as spatially averaged electric field strength (*E*_ave_), defined as the average electric field strength over the length of a single pumping unit (detailed described in Supporting Information Simulation in COMSOL Multiphysics section). **Figure** **3**d,e shows the good agreement between calculated *E*_ave_ and pumping velocity in different devices.

## 4 Active Microfluidic Devices Based on Asymmetrically Coated Microelectrodes Pump

As stated previously, our active pumping device is much simpler than the previously demonstrated planar ACEO device,^[^ ^26^ ^]^ which can potentially be scaled up for large‐scale integration. Here, we demonstrate a microvortex generator and multichannel microfluidics based on our active pump.

In microfluidic lab‐on‐chip system, the intrinsic laminar flow nature makes the efficient mixing of fluid challenging for many applications.^[^ ^39^ ^]^ In particular, the generation of vortex at microscale proved to be a key technique for a range of important tasks, including cell sorting, cancer cell enrichment, particles separation, and micromixing.^[^ ^40^, ^41^, ^42^ ^]^ For our microelectrode pump, as the pumping direction can be easily controlled with deposition angle, the pumping flow field can be designed with high precision. Specifically, the triangle‐ and square‐shaped vortex generation units with 100 μm in side length are fabricated and tiled on a substrate surface ≈2 mm^2^ in size, which creates a macroscopic vortex generator.

As illustrated in Figure 3a, by changing the deposition sequence, a clockwise or counterclockwise vortex unit can be fabricated on the triangle and square vortex layout (see Fabrication of Microvortex Generator, Supporting Information). The right part in Figure 3b indicates the different deposition directions of the gold electrode on a single vortex unit, which will form a clockwise vortex flow on it. When plenty of vortex units are assembled, a microscopical vortex pattern could be generated as the experiment vorticity field indicated in Figure 3c, where a clockwise vortex is surrounded by other 3 (for triangle) or 4 (for square) counterclockwise vortexes. This scalable microvortex generation device offers a prototype for studying flow patterning and suggests the possibility of being used as a micromixing chip.^[^ ^10^, ^43^, ^44^, ^45^, ^46^ ^]^

**Figure 4:**
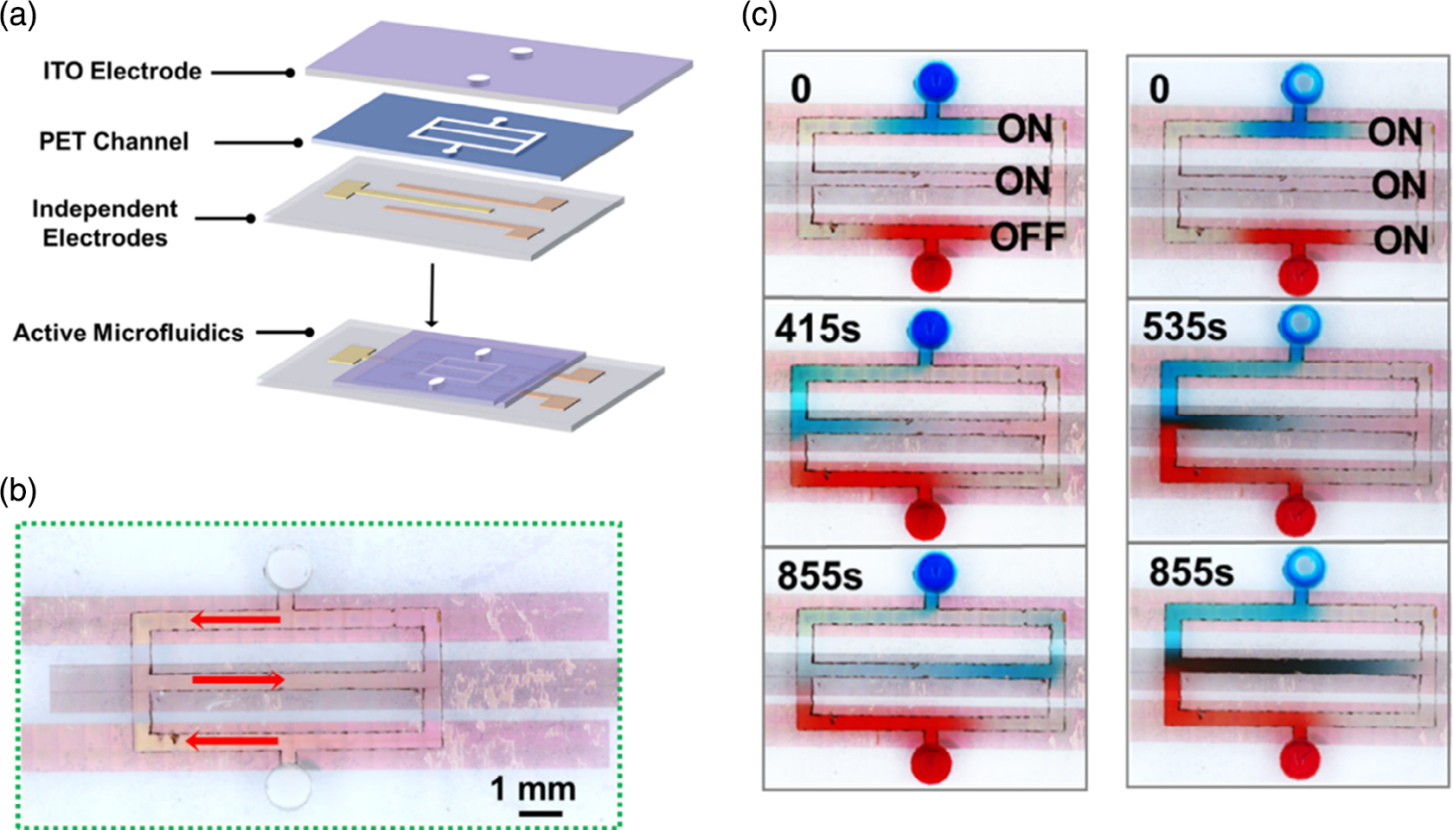
Demonstration of independently controlled three channels microfluidics device. a) Schematics of the sandwiched structure of the microfluidics device. b) The microfluidic circuit covering on three independent electrodes. Fluid can be injected from the top/bottom channels. The red arrows indicate the flow direction in the channel when powering on all electrodes. c) Partial photographs over 855 s when upper and middle electrodes are activated (left), and all electrodes are activated (right), snapshotted from Video S3, Supporting Information.

In addition, as these pumping devices are powered by AC power without any external moving part, they are suitable for miniaturization and integration for lab‐on‐chip applications.^[^ ^4^, ^5^, ^47^ ^]^ Here, we further demonstrated an active microfluidic device with independently controlled flow channels, which is highly desired in microanalysis chip. As shown in **Figure** **4**a, the device is composed of three parts, ITO cover glass, microchannel layer in PET film, and bottom slide with three individual asymmetric pumping electrodes (see Supporting Information for detailed fabrication procedure).

The pumping circuit is designed to have three microfluidic connected channels that cover each electrode individually. The upper and lower pumping electrodes are layered with the pump direction from right to left, and the middle pumping electrode is layered in the opposite direction. The fluid can be injected from the top/bottom channel, as shown in Figure 4b. Food dyes were used to color the fluid in the microfluidics devices to elucidate the active flow. As shown in the left of Figure 4c, when only the top and middle pumping electrodes are activated, the directional flow in top channel shows faster flow than the lower channel as the blue solution flows in the middle channel before the red solution. When all pumping electrodes are activated, both blue and red solutions are pumped into the middle channel simultaneously and mixed into green solution (right of Figure 4c), demonstrating the excellent controllability of our active microfluidic device at macroscopic scale. After a simple calculation, we found that the flow rate in the channel is around 5.5 nL s^−1^. Compared with other works listed in Table S3, Supporting Information, our microfluidic device's pumping rate is comparable to some other ACEO‐based microfluidic devices, such as the ACEO‐based bubble‐assisted microfluidics devices developed by Li et al.^[^ ^48^ ^]^ However, it is much lower than other microfluidics devices actuated by other mechanisms. This suggests that our design is more suitable for creating complex flow fluid due to its simplicity rather than pumping large volumes of liquid.

## 5 Conclusion and Outlook

We have presented a unidirectional micropumping system with asymmetrically structured microelectrodes. The spatial asymmetry of the electrode bends the local electrical field, and then electroosmotic slip flow generates because of the Coulomb interaction between the tangential electric field and the induced electric double layer. We showed that pumping performance is dependent on the electrode structure by numerical simulation and experimental velocity profile.

Due to the simple structure and fabrication, it is possible to precisely generate any desired flow pattern on a microscopical or macroscopical scale. Finally, we demonstrated a microvortex device by combining pumping units in different pumping directions. An active microfluidics device demonstrated the macroscopical control of the flow field. This micropump device can be easily scaled up by applying nanoimprint or nanomoulding techniques for cheap and fast fabrication, which may be applied to active mixing processes and fabrication of lab‐on‐chip devices.

## Conflict of Interest

The authors declare no conflict of interest.

## Supporting information

Supplementary Material

## Data Availability

The data that support the findings of this study are available from the corresponding author upon reasonable request.
