# Supplementary material for: Simple Electroosmotic Pump and Active Microfluidics with Asymmetrically Coated Microelectrodes
Source: Small Sci. 2023 Jul 9;3(9):2300026. doi: 10.1002/smsc.202300026 (PMC11935900; doi:10.1002/smsc.202300026)
Supplement: Supplementary file 1 — Supplementary Material [file SMSC-3-2300026-s001.zip › SI-non-highlighted.pdf]

Supporting Information

Simple Electroosmotic Pump and Active Microfluidics with Asymmetrically Coated Microelectrodes

Jun Liu[1], Jiawei Chen[1], Jia Dai[1], Jinyao Tang[1*]

[1]Department of Chemistry, The University of Hong Kong, Pokfulam 999077, Hong Kong.

[*]e-mail: jinyao@hku.hk

1. Device Fabrication and Characterization

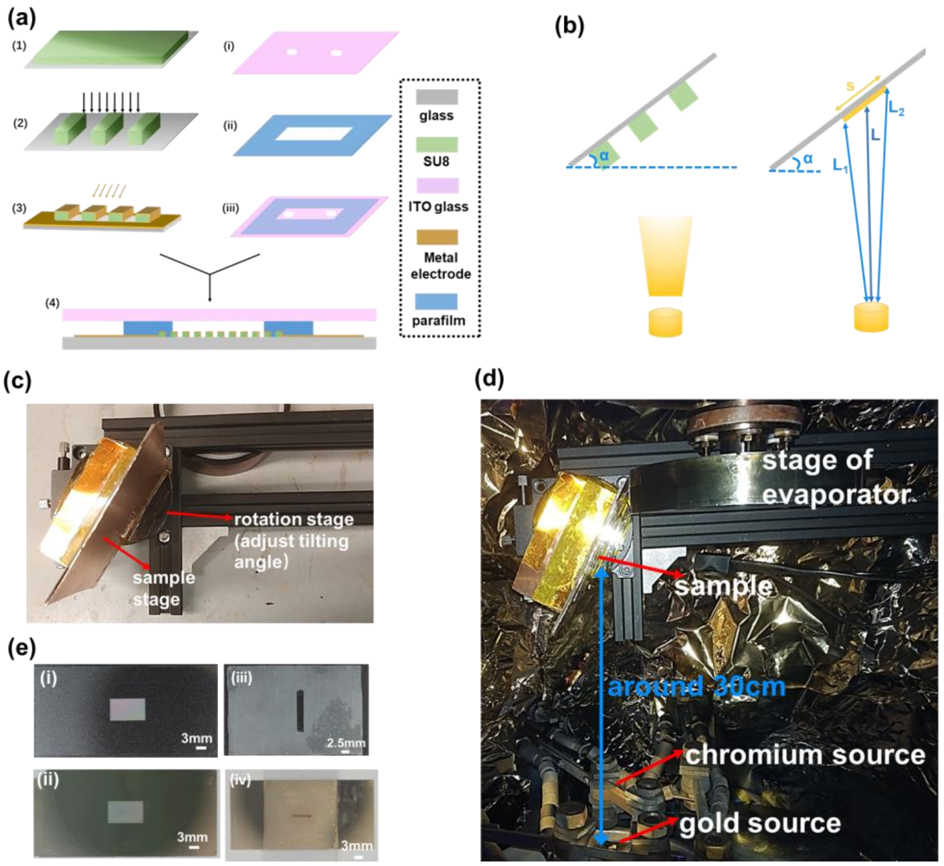

Figure S1. The micropumping device fabrication. (a) The schematic of the microfabrication process. 1. spin-coating of ~1 μm layer of SU8 on the glass substrate. 2. Generating line SU8 patterns by E-beam lithography. 3. Depositing gold electrode on the patterns by GLAD method. 4. Thermally bonding ITO glass to the bottom electrode glass by the middle layer of parafilm under heating. (b) The GLAD method for asymmetrically coating of gold. The angle α is the tilting angle. The s is the length of microfluidic channel (around 10mm) covered on the bottom electrodes. The L, L_1 and L_2 are the distance between the center, lower endpoint and top endpoint of s and the gold source, respectively. L is fixed at 30cm. (c) A sample platform with a sample stage for holding samples and a rotation stage to adjust the tilting angle for different samples. (d) The experimental set-up for depositing gold electrodes on the SU8 pattern in the chamber of thermal evaporator with a built-in stage of evaporator, the sample platform, chromium source and gold source. (e) The photographs of glass with SU8 pattern (i), after deposition of gold (ii), an ITO glass covered with a layer

of parafilm (iii) and a typical device used in the velocity observation (iv).

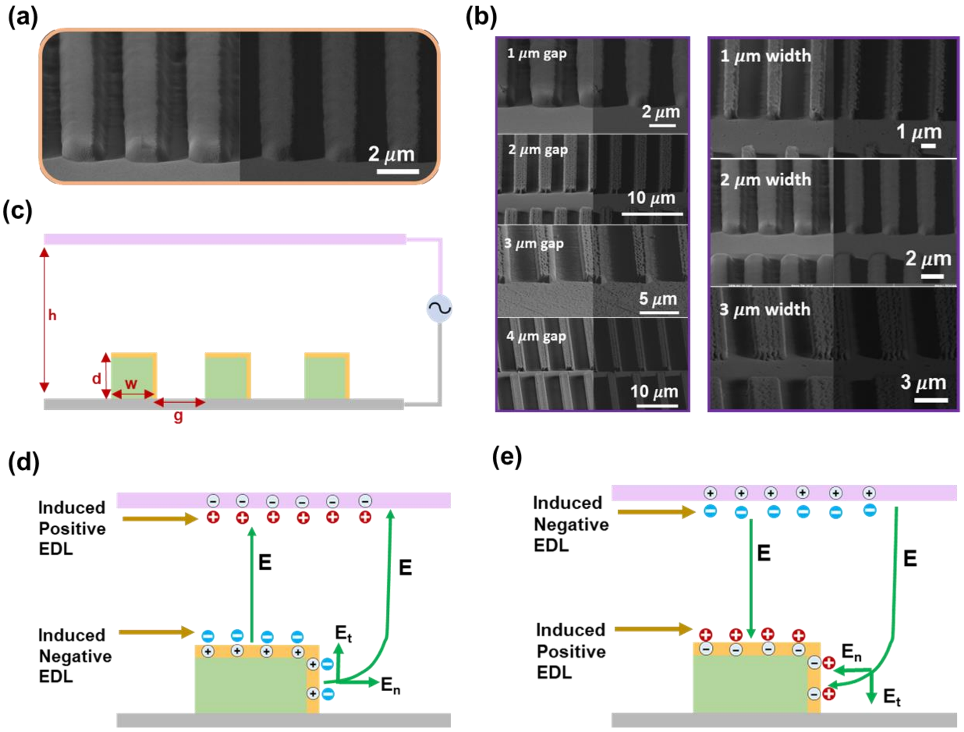

Figure S2. The micropumping device characterization and formation of electric double layer (EDL). (a) Original SEM image of false-colored SEM in Figure 1b. The right part of (b) is taken under In-Beam BSE mode, where the bright area indicates the cover of gold. (b) SEM images of the devices with different gap distances (1 μm, 2 μm, 3 μm, 4 μm, the width is 2 μm) and different widths (1 μm, 2 μm, 3 μm, the gap distance is 2 μm). The right part of the images is taken under In-Beam BSE mode where the bright area indicates the cover of gold. (c) Side view of the device. The w, d, g and h are the geometry parameters of the device, width of line electrode, depth of line electrode, gap distance between line electrodes and the flow channel height, respectively. (d) During the positive cycle of applied AC signal, a negatively charged EDL is created on the positively polarized bottom gold electrodes under applied electric field (green line) while the top ITO electrode generates the cation-rich EDL. (e) During the negative cycle of applied AC signal, a positively charged EDL is created on the negatively polarized bottom gold electrodes under applied electric field (green line) while the top ITO electrode generates the anion-rich EDL.

Figure S1 shows the typical fabrication process of a micropumping device and a photography of an actual. We used an ordinary borosilicate cover glass (24mmX50mm) with a 0.13-0.16mm thickness as the substrate. The SU8 line pattern was fabricated using electron beam (E-beam) lithography. A 1 μm-thick layer of diluted SU8 2000 was deposited on the glass by spins-coating for 60 s at 4000 rpm with a 5 min prebake process at 95 ℃. The depth d of the line electrode was 1 μm for all the devices in our work unless otherwise stated. The micropattern was exposed under E-beam controlled by Nanometer Pattern Generation System (NPGS) in SEM. Then the glass was post-exposure baked for 5 mins at 95 ℃ and developed for around 1 min in propylene glycol methyl ether acetate (PGMEA) solvent. A hard bake

process was conducted at 180 ℃ for 30 min to cure the SU8 pattern. Before the deposition of electrode, around 10 nm silica was deposited on the wafer by Magnetron Sputtering System. A 5 nm thick adhesion layer of chromium and a 30 nm thick layer of gold was asymmetrically deposited on the pattern using the glancing angle deposition (GLAD) method in a thermal evaporator under $5 \times 10^{-5}\, Pa$ to $2 \times 10^{-4}\, Pa$. The gold covering area on the pattern was confirmed under In-Beam BSE mode of SEM, by which the pattern coated with gold was bright.

To ensure the reproducibility of the micropump device, we designed a sample platform (Figure S1 c) to hold the glass with SU8 patterns. It can be fixed on the built-in stage in the chamber of the thermal evaporator, which ensures the same relative location of the sample and the gold source during the fabrication of different devices (Figure S1 d). In addition, the rotation stage on the sample platform can be used to adjust the tilting angle for devices with different geometry parameters.

This glass decorated with SU8 pattern and gold electrode served as one connection pad for applying ac potential. Another counter connection pad was a piece of 150 nm thick ITO glass. Two holes with a diameter of 1 mm were drilled into the ITO glass with a distance of 10 mm that was used for the injection of solution. Then a layer of parafilm cut with desired channel shape was sandwiched between the bottom electrode pattern and ITO glass by thermally bonding on the hotplate at around 100 ℃ for 10 min.

For tunable gap distance and width (Figure S2 b), the devices' fabrication method is the same as in the previous process, and gap or width is adjusted by defining the pattern file in NPGS software. To keep the same gold covering structure, the tilting angle during thermal deposition of the electrode by GLAD method is different for the devices with different widths and gaps as shown in the Table S1. To avoid the affecting pump performance due to gradient deposition during the coating gold electrode, only the central region with 10mm length and 1mm width of the electrode covering area (around 14mm length and 7mm width) is used as the bottom of microfluidic channel (s in the Figure S1 b). As the deposition thickness of gold is negatively proportional to the distance between the sample and gold source, the difference in gold thickness at two endpoints of s can be calculated with s of 10mm, L of 30cm, gold thickness of 30nm at the center of s and a given tilting angle α. The results in Table S2 indicate a maximum difference of gold thickness at the fluidic channel is less than 1nm, which will not alter the pump

performance.

Electrode geometry (w,g,d)/μm	Tilting angle (α)	Difference of gold thickness at the fluidic channel/nm
(2,1,1)	45°	0.71
(2,2,1)	63°	0.89
(2,3,1)	71°	0.94
(2,4,1)	76°	0.97
(1,2,1)	63°	0.89
(3,2,1)	63°	0.89

Table S1. The tilting angle and difference of gold electrode thickness for different devices.

2. Experimental Setup and Speed Calculation

The sinewave ac signal from a function generator was applied to the bottom gold electrode and the ITO ceiling of the device. Besides, an oscilloscope was connected to the device to monitor the sinewave. The set-up was placed on a laser scanning confocal microscope (LSCM) to observe the flow motion on this device, as shown in Figure S3 a. We used 500nm silica particles synthesized in our lab to trace the flow. We only used the fluorescent mode of LSCM to record the motion of tracers under white light illumination.

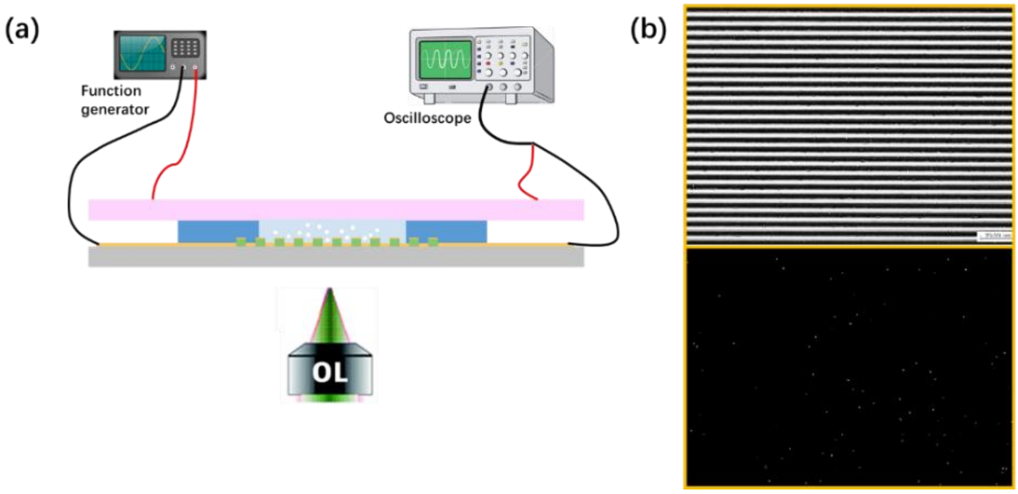

Figure S3. (a) Illustration of the experiment set-up for observation the fluid motion on different micropumping devices. (b) Removal of background of the one frame snaped from the particle tacking video. The bottom image showed the clear tracer particles (white spots) after removing the background.

The obtained videos were composed frame by frame into grayscale pictures. There are

many bright and dark stripes on the frames because of the shutter effect of the half-coating electrode on the glass substrate. Hence, these pictures were further processed to remove the background using *PIVlab,* as shown in Figure S3 b. After removal of the bright and dark stripes, the flow velocity was tracked by the *ImageJ*. The velocity was at least 100 tracer particles' average velocity tracked from a video of ~20 s. The error bar was obtained by calculating the confidence interval at 95% confidence for these particles.

micropump	Applied voltage (V_{peak})	Distance between paired electrodes(μm)	Electric field (V/μm)	Velocity (μm/s)	$\frac{Velocity}{(electric\ field)^2}$ (μm/s/V^2)
IDE-based ACEO pump[1]	1.13	4.5 and 15.6	0.25 and 0.07	60	960 and 12245
IDE-based ACEO pump[2]	0.5	3 and 8	0.17 and 0.06	18	622 and 5000
IDE-based ACEO pump[3]	1.41	2 and 6	0.71 and 0.24	150	298 and 2604
High step ACEO pump[4]	1.5	4.5 and 15.6	0.33 and 0.10	420	3856 and 42000
Our micropump (1μm width, 2μm gap, 1μm depth)	1.0	30	0.03	29	32222

Table S2. The pumping performance of our micropump compared with previous ACEO-based micropumps.

3. Simulation in COMSOL Multiphysics

The numerical simulation in this work was calculated by *COMSOL Multiphysics 5.4* software package. The 3D structure pattern was simplified as a 2D physics model in *COMSOL Multiphysics* by taking a side slide from the devices in the side view. In other words, the flow channel was regarded as a rectangular domain filled with water. The width of the rectangular domain was set to be 50 μm to minimize the simulation time (the real channel's width is ~1 mm). The height of the rectangular domain was 30 μm with, the same size as the real channel's height. The bottom side of the rectangular domain was replaced by a periodically raised step with the same width, depth, and gap of the real line SU8 pattern. As for the planar electrodes

device simulation, the depth of the bottom periodic raised step was changed from 1 μm to 30 nm (the thickness of the gold electrode), and the rest structure was kept the same.

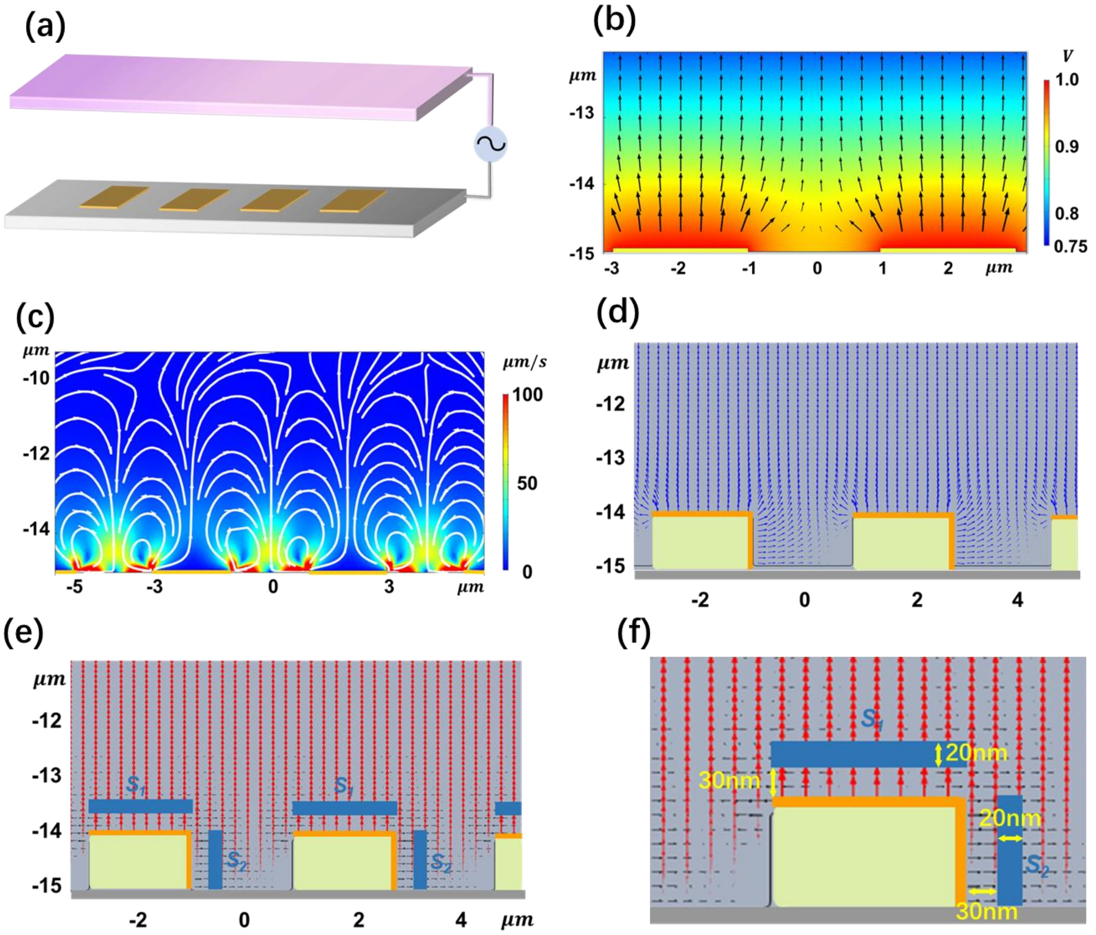

Figure S4. (a) the schematics of the planar electrode device used in the control experiment. Simulated results of symmetrical electric field (b) and flow field (c) near the planar electrode surface. The black arrows indicate the electric field, and the color scale indicates the voltage in (b). The white arrows indicate the flow direction, and color scale indicates the velocity in (c). The geometry parameter h, w, d and g in the simulation is 30 μm, 2 μm, 1 μm and 2 μm. The AC amplitude is 1 V_{peak} and frequency is 4000 Hz. (d) Simulated electric field on device with 2 μm linewidth and 2 μm gap, blue arrows indicate the electric field. (e) The x-axial component (black arrows) and y-axial component (red arrows) of the electric field in the (d). The blue block is the selected area *S* for calculating average electric field strength. (f) The partially enlarged view of (e). For (d), (e) and (f), the green and yellow block represent the SU8 and gold covering area, respectively.

The spatial averaged electric field strength (E_{ave}) is defined as the average electric field strength near the slip-generated surface over the length of single pumping unit. Firstly, the local region was defined as a small rectangular area *S* with 20nm width paralleled with the gold covering surface at a distance of 30nm away from the gold surface (see Figure S4 e). Then, the average mean of the electric field strength in this local region, $E_{S,average}$ was obtained by

calculating the surface average value of electric field component paralleled with the gold surface, E_{para}, using this equation $E_{S,average} = \frac{1}{s}\iint E_{para}(S)dS$ in COMSOL Multiphysics, where S is the area of region *S*. For example, for region S_1 in Figure S4 e, E_{para} is the x-component of the electric field. Then, the spatial averaged electric field strength (E_{ave}) was calculated by $E_{ave} = \frac{E_{S,average}}{w+g}$, where *w* and *g* are the width and gap of the step in the simulation model, respectively.

4. Fabrication of Microvortex Generator

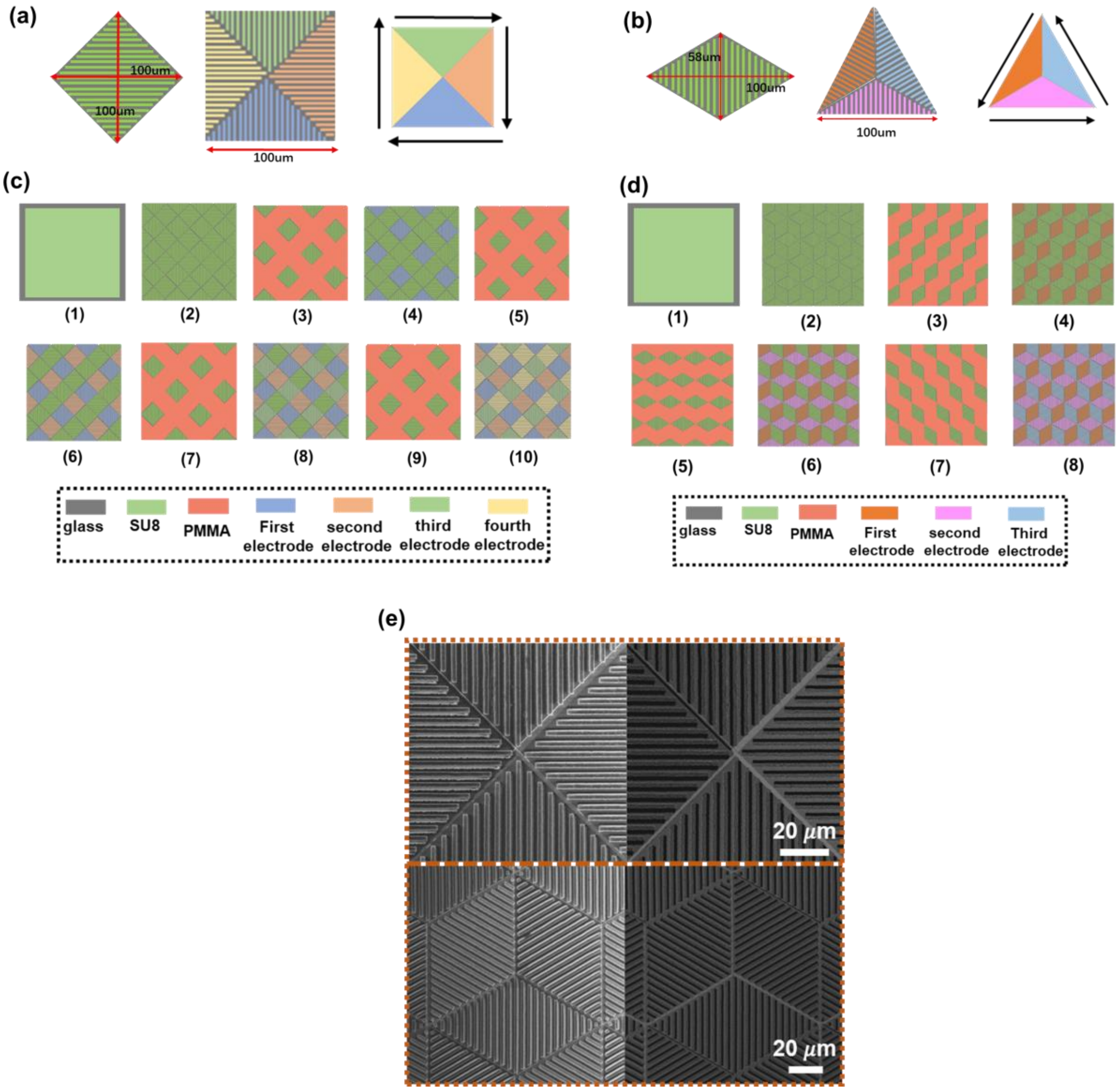

Figure S5. The schematic of the microfabrication process for microvortex generation device. (a) and (b) shows the size of SU8 pattern unit and microvortex unit for square vortex and triangle vortex, respectively. (c) The main steps for the fabrication of square vortex generation device. 1. spin-coating a ~ 1 μm thick layer of SU8 on glass slide. 2. SU8 microstructure array by E-beam lithography. 3. Unmasking the region for first electrode deposition using PMMA. 4.

After depositing the first electrode. 5. Unmasking the region by PMMA. 6. After depositing the second electrode. 7. Unmasking the region by PMMA. 8. After depositing the third electrode. 9. Unmasking the region by PMMA. 10. After depositing the fourth electrode. (d) The main steps for the fabrication of triangle vortex generation device. The process is basically the same with the steps in (c). (e) Original SEM image of false-colored SEM in Figure 3b. The right part of (e) is taken under In-Beam BSE mode, where the bright area indicates the cover of gold.

We used the E-beam alignment lithography method to locally tune the direction of the electrode on the SU8 pattern. The size of the square and triangle vortex flow is indicated in Figure S5 a and b, respectively. The final flow generated region on the device is mm-scale by arraying small μm-scale SU8 pattern units. The basic μm-scale SU8 pattern unit is composed of line microstructure with different lengths but the same width (2 μm) and gap (2 μm). The side length of the square SU8 pattern unit is 70.7 μm with diagonal of 100 μm. Four square SU8 pattern units with different electrode deposition directions are assembled. Their diagonals form a new square pattern with side of 100 μm. This square pattern is the basic microvortex unit. The situation is same for the triangle microvortex unit.

Each quarter (for square vortex or trisection for triangle) of the microvortex unit offers a flow vector along the side of the square (or triangle). A clockwise or counterclockwise direction vortex will generate on this unit when connecting these vectors by head to tail. By controlling the vorticity status of each vortex unit, different microscopical flow patterns can be formed. The detailed fabrication procedures are illustrated in Figure S5 c and d.

A ~1 μm thick layer of diluted SU8 2000 was deposited on the glass by spin-coating for 60 s at 4000 rpm with 5 min prebake process at 95 ℃. Then, several hundreds of square μm-scale SU8 pattern units were arrayed on the glass with alignment makers by E-beam lithography. Afterwards, four times electrode deposition processes were carried out in turn. We used PMMA as the mask layer to uncover the area for electrode deposition on SU8 pattern by E-beam lithography. The alignment process was also done in the NPGS using the pre-written makers. Then we deposited the first electrode on the uncovered area, followed by lift-off process. This process was repeated on the remaining area for deposition of the second, third, and fourth electrodes. For the latter electrode deposition process, each time wafer was rotated 90° for the following electrode deposition to generate the different flow directions on different electrodes. For the triangle microvortex, the total fabrication was nearly the same with the square microvortex. The only difference was that it only needed three times electrode

deposition processes with 120° rotation angle of wafer between two electrode deposition processes.

5. Fabrication of Microfluidics Device

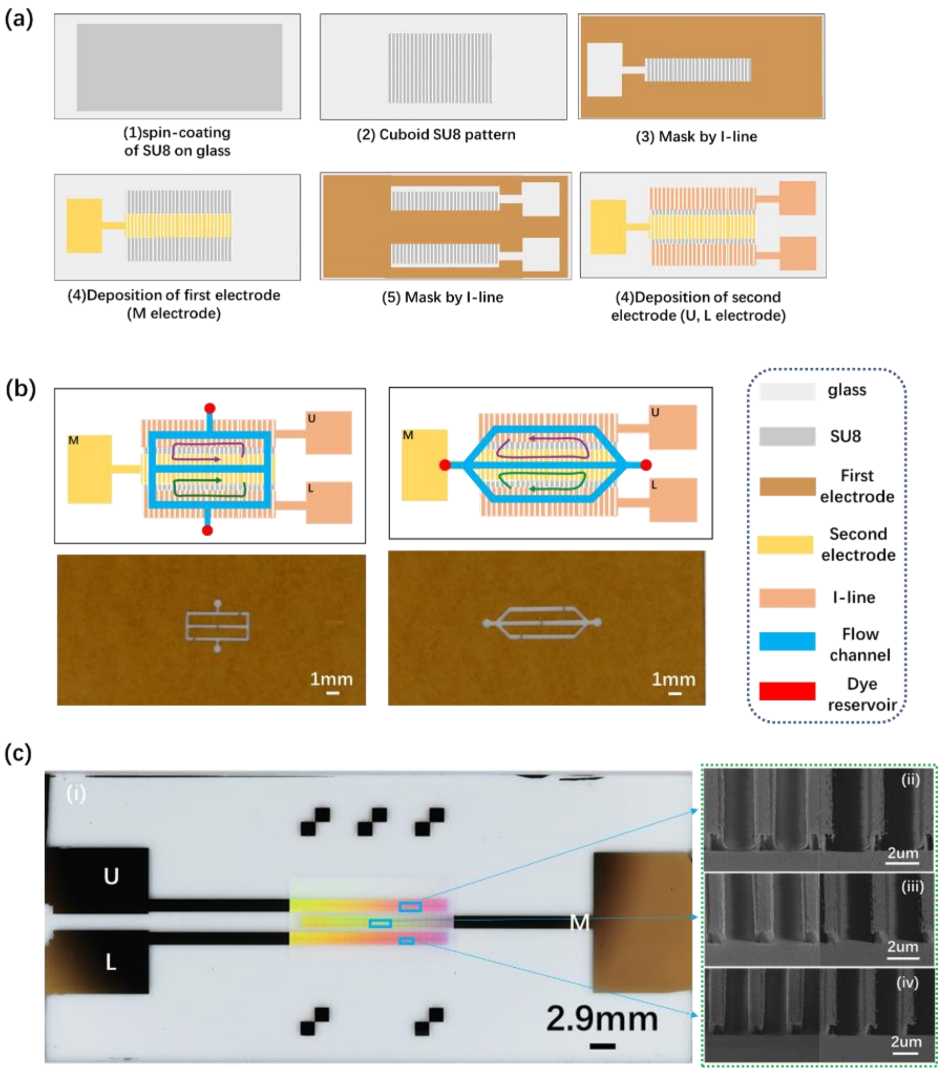

Figure S6. The microfluidic device fabrication. (a) The illustration of fabrication process for bottom electrode. 1. spin-coating of SU8 on a glass slide. 2. Line SU8 microstructure array on the slide by E-beam lithography. 3. Unclosing the region using I-line by photolithography method. 4. After depositing of the first electrode (M electrode). 5. Unclosing the region using I-line. 6. After depositing the second electrode (U and L electrode). (b) Schematics (upper) of the top view of two microfluidic circuits and the photographs of the PET microchannels (bottom). Three channels can be individually driven by three separate electrodes (U, M and L). The upper flow circulation (red lines) forms when the electrode M and L are powered on, same for the lower circulation (green lines). Two circulations with opposite direction of flow are generated when three electrodes are powered on. (c) Photography of bottom SU8 pattern deposited with gold on the glass substrate (i). The SEM images (ii, iii, iv) indicate the opposite direction gold electrode on the SU8 pattern, which results in the opposite flow direction of two circulations. The right part of SEM image is taken under In-Beam BSE mode where the bright area indicates the cover of gold.

First author and Ref.	Actuation mechanism	Structure	Chamber and size	Power	Flow rate
Ashourl[5]	MHD-DC	PDMS	Φ 2 mm	6V	700uL/min 12245
Russel[6]	EDH-DC	PDMS-Glass	5mm×100μm	700V	0.47mL/min
Jiang[7]	DCEO	PDMS	no	200V	240μm/s
Piñón[8]	ACEO	Polymer	Planner	12V,1kHz	29.03 μm/s
Li[9]	ACEO	PDMS-Glass	80μm (Width), 50μm (Height)	60V	500pL/s
Our microfluidics device (1μm width, 2μm gap, 1μm depth)	ACEO	PET film-Glass	300μm (Width), 30 μm(Height)	1V,4kHz	5.5nL/s

Table S3. Summary of properties for some micropumps driven by electric field.

We fabricated a three-channel microfluidics device with three independently controlled electrodes on the bottom. The width of the microfluidics channel was designed to be ~300 μm. Parafilm is unsuitable for such narrow channels because it’s easily fusible and deformed under heating. The thermal bonding process will destroy the microchannel if we use parafilm. Therefore, we used another material to replace parafilm, polyethylene terephthalate (PET) film that can be easily cut into the desired shape by laser cutter.

The whole fabrication process for the microfluidic was indicated in Figure S6 a. The flow direction in different channels was controlled by changing the deposition direction of electrode for different channels. Firstly, cuboid SU8 pattern with 1 μm width, 2 μm gap and 1 μm depth was arrayed on a galss slide by E-beam lithography. Then we deposited the first electrode on the SU8 pattern by using photolithography to unmask the deposition region. A layer of I-line photoresist was spin-coated on the pattern for 40 s at 4000 rpm. The region for depositing the first electrode was exposed under UV illumination and unmasked after the development,. In this process, alignments markers for second electrode deposition were also coated on the surrounding of the electrodes during the thermal evaporation process. Next, a second photolithography process was used to uncover the region for the second electrode position. The alignment was done during the exposure of I-line to avoid the connection betwteen first and second electrodes. The wafer was rotated for 180° during the second thermal evaporation

process resulting in the opposite direction of electrodes and consequently reverse flow direction on these electrodes. Finally, the ceiling ITO glass and the bottom slide pumping electrodes were assembled together by the middle layer of PET film (see Figure S6 b) with a designed microchannel.

6. Reference

[1] A. B. Brown, C. G. Smith, A. R. Rennie, *Phys. Rev. E: Stat Nonlin Soft Matter Phys.* **2001**, 63, 016305.

[2] V. Studer, A. Pépin, Y. Chen, A. Ajdari, *Microelectron. Eng.* **2002**, 61-62, 915.

[3] M. Mpholo, C. G. Smith, A. B. D. Brown, *Sens. Actuators B* **2003**, 92, 262.

[4] J. P. Urbanski, T. Thorsen, J. A. Levitan, M. Z. Bazant, *Appl. Phys. Lett.* **2006**, 89.

[5] M. Ashouri, M. B. Shafii, A. Moosavi, *J. Micromech. Microeng.* **2017**, 27, 015016.

[6] M. K. Russel, S. M. Hasnain, P. R. Selvaganapathy, C. Y. Ching, *Microfluid. Nanofluid.* **2016**, 20, 112.

[7] H. Jiang, N. Fan, B. Peng, X. Weng, *Appl. Phys. Lett.* **2017**, 110, 184102.

[8] M. Vázquez Piñón, B. Cárdenas Benítez, B. Pramanick, V. H. Perez-Gonzalez, M. J. Madou, S. O. Martinez-Chapa, H. Hwang, *Sens. Actuators A* **2017**, 262, 10.

[9] X. Li, S. Liu, P. Fan, C. F. Werner, K. I. Miyamoto, T. Yoshinobu, *Sens. Actuators B* **2017**, 248, 993.
